# Exploring the Impact of Safer Sports Betting Promotion on Social Media: An Experimental Study

**DOI:** 10.1007/s10899-024-10280-z

**Published:** 2024-01-19

**Authors:** Scott Houghton, Mark Moss

**Affiliations:** Department of Psychology, Northumbria University, Newcastle-upon-Tyne, NE1 8ST UK

**Keywords:** Gambling, Harm-reduction, Social media, Intervention

## Abstract

The current study assessed the effectiveness of delivering safer gambling messaging to regular sport bettors on social media. It also assessed whether the content of the message impacted message effectiveness. A 3 × 2 mixed factorial design was employed, with 281 participants randomly assigned to follow one of three Twitter accounts. The accounts either sent out informational messages, self-appraisal messages or emotional self-efficacy messages. Participants reported betting behaviour from the two weeks prior to following the accounts, in addition to during the two-week intervention period, using information from their online gambling accounts. Participants reported readiness to change gambling behaviour pre and post the two-week intervention period. A significant main effect of intervention stage highlighted reductions in betting behaviour and increased readiness to change behaviour. However, there was no significant main effect of condition and no significant interaction between intervention stage and condition upon betting behaviour or readiness to change. The findings suggest receiving safer gambling messages on social media may lead to a reduction in betting behaviour. However, similar reductions in behaviour were observed in the experimental and control conditions. Further research is needed to clarify whether changes in behaviour observed in the current study would extend over a longer period of time.

**Supplementary Information:**

The online version contains supplementary material available at 10.1007/s10899-024-10280-z.

## Introduction

There is a need for the development of new safer gambling strategies to reduce the harm caused by gambling within Great Britain. Many currently used safer gambling strategies lack a strong empirical evidence base for effectively reducing gambling harms (McMahon et al., 2019). Building upon this, a recent qualitative study of young adult gamblers in the UK described how they felt that safer gambling messages within marketing were not very effective due to being tokenistic (Torrance et al., 2020). Whilst many current safer gambling strategies focus on distributing messages to gamblers during active gambling sessions, such strategies may be limited due to the high emotional states present during gambling sessions (Sohn et al., 2015) that may push bettors away from rational decision making. As such, there is a need for further research exploring the effectiveness of safer gambling messaging delivered outside of active gambling sessions.

One potential avenue for safer gambling messaging is to deliver it through social media. Previous research in other areas of public health, such as smoking cessation and cancer prevention, has provided evidence of social media interventions having high reach to target audiences (Gough et al., 2017) and being able to produce attitudinal and behavioural change (Laranjo et al., 2015; Naslund et al., 2017). Previous messaging-based interventional health campaigns have advocated for the use of the platform Twitter over other social media networks. This is due to the website’s greater open accessibility, wide-reaching capabilities for information distribution and its functionality for allowing researchers to pre-schedule the postings of timed messages directly onto users’ content feeds (Pechmann et al., 2015). In comparison to traditional approaches which assess the effectiveness of safer gambling techniques only at the moment of active play, it is hoped that the integration of safer gambling campaigns directly into the social media feeds of bettors would instead lead to a more ‘prolonged’ user exposure to the intervention over time. Safer gambling messages could then be successfully implemented through their embedding into day-to-day online activities, behaviours and rituals of those gamblers who are most at risk.

Nevertheless, an important consideration within any safer gambling message is how best to frame such messaging to produce positive changes in behaviour. Informational messages that aim to increase a gambler’s knowledge of odds and probabilities have been used previously within safer gambling campaigns (Blaszczynski et al., 2011). The rationale for using informational messaging revolves around the idea that risky gambling behaviour is the result of erroneous cognitions around gambling and therefore correcting such beliefs should enable gamblers to regulate their own behaviour (Monaghan et al., 2009). However, previous research has consistently shown that providing gamblers with informational messages during active gambling sessions has no impact upon behaviour (Cloutier et al., 2010; Gainsbury et al., 2015; Monaghan & Blaszczynski, 2010). This has led researchers to explore alternative types of messaging.

Self-appraisal messages are one such type of message and are framed in a way which invites gamblers to self-reflect upon their own behaviour in order to make the messages more personally relevant. These have been shown to have a greater impact upon gambling behaviour (Gainsbury et al., 2015; Harris & Parke, 2016; Monaghan & Blaszczynski, 2010), likely due to increasing autonomy within decision making (Pavey & Sparks, 2010) and through increasing self-awareness of gambling behaviour (Harris & Griffiths, 2017). A further argument put forward for the use of self-appraisal messaging is that it may help address some of the commonly observed contributors towards problematic behaviour. Harris and Griffiths (2017) highlight that messages which directly attempt to get gamblers to raise their self-awareness of a behaviour may help prevent gamblers entering dissociative states, something which has been shown to correlate with disordered gambling behaviour (Stewart & Wohl, 2013). However, research has shown self-appraisal messages are not effective when bettors are in a winning position. This could be problematic as gamblers may be gambling in a risky manner but winning enough in the short-term to not perceive the messages as relevant. A further limitation of the current evidence base for using self-appraisal messages is the fact there is only support for using such messages within active gambling sessions. Therefore, research is needed to investigate whether findings can be replicated when messages are delivered outside of gambling sessions.

Another type of messaging consistently used within the wider addiction literature, which is particularly good at capturing attention, is emotional messaging (Hammond, 2011; Harris et al., 2018). Fear appraisals are the most used type of emotional messaging, based upon the belief that individuals are motivated to protect themselves when they feel endangered (Janis, 1967; Rogers, 1975). Whilst some evidence exists supporting the effectiveness of fear appeals in areas such as binge drinking, smoking and gambling (Carrera et al., 2010; Munoz et al., 2013; Wang et al., 2015), other research suggests their effectiveness is low in high-risk populations (De Vos et al., 2017) and when self-efficacy is low (Peters et al., 2013). Therefore, in order to develop safer gambling strategies which benefit those across the spectrum of gambling-related harm - there is a need for other types of emotional messaging which promote self-efficacy to be explored. This is particularly the case for sports betting, which has received comparatively little attention within academic literature on harm reduction messages. This is despite the high levels of gambling integration within sporting contexts and growing concerns around the impacts this may have on both current and future sports bettors (Hing et al., 2023). As such, there is a particular need for research to consider what safer gambling messaging is most effective within a sports betting context.

One example of such an approach to safer sports gambling promotion can be seen within the recent ‘BetRegret’ campaign being run by GambleAware, the leading independent gambling charity in Great Britain. The campaign had a media spend of £3.1 million and aimed to provide bettors with safer gambling advertising that was both emotionally stimulating and clearly distinct from betting advertisements (GambleAware, 2020). For the first stage of the campaign, several short advertisements were created which were framed around situations where bettors may subsequently regret their betting decisions (e.g. betting when drunk or intoxicated). As such, the enlisted messages of the campaign took a preventative approach, whereby bettors were encouraged to identify with the emotion of regret often felt in those situations and to therefore avoid such behaviour in the future. Initial evaluation of the success of the first stage of the campaign demonstrated that bettors self-reported increased thoughts about behaviour change (GambleAware, 2020). They also reported making positive changes to their gambling behaviour, specifically on the behaviours highlighted within the campaign. Whilst this offers some initial evidence of the effectiveness of the type of messaging used within the campaign, further evaluation is required to assess its effectiveness in comparison to other types of safer gambling messaging. There are also concerns about the role of memory biases in asking people to report upon changes to their betting behaviour (Auer & Griffiths, 2017). Therefore, any future evaluation of the campaign should take this into consideration and aim to measure actual gambling behaviour.

### Aims and Hypotheses

The impact of safer gambling messaging during active gambling sessions may be limited due to the high emotional states present during gambling sessions (Sohn et al., 2015). As such, the current study first aims to assess the effectiveness of delivering safer gambling messages to sports bettors through social media. Given the mixed evidence base for the effectiveness of different types of messaging (Cloutier et al., 2010; Gainsbury et al., 2015; Peters et al., 2013), the second aim of the current study is to assess which is the most effective type of safer gambling message to deliver on social media.

Based upon the literature presented here, the following predictions have been made:

#### H1

It is predicted that bettors in both the self-appraisal messages and emotional, self-efficacy messages conditions will show an increased readiness to change gambling behaviour after receiving safer gambling messages on social media for 14 days, whilst those in the informational messages condition will not.

#### H2

It is predicted that bettors in both the self-appraisal messages and emotional, self-efficacy messages conditions will show reductions in their betting behaviour during the 14 days where they are receiving safer gambling messages on social media compared to the 14 days prior to receiving the messages. No changes in betting behaviour are expected for those in the informational messages condition.

## Method

### Design

The current study employed a 3 × 2 mixed factorial design. The independent groups factor was experimental condition with three levels: informational messages, self-appraisal messages and emotional/self-efficacy messages. Informational messages acted as a control condition, given previous research has demonstrated they do not impact gambling behaviour (Cloutier et al., 2010; Gainsbury et al., 2015; Monaghan & Blaszczynski, 2010). The repeated measures factor was experimental stage with two levels: pre-intervention and post intervention. The dependent variables were readiness to change gambling behaviour and three measures of sports betting behaviour over the past 14 days, including: number of bets placed, number of days where participants placed a bet and their total money staked.

### Participants

**Fig. 1:**
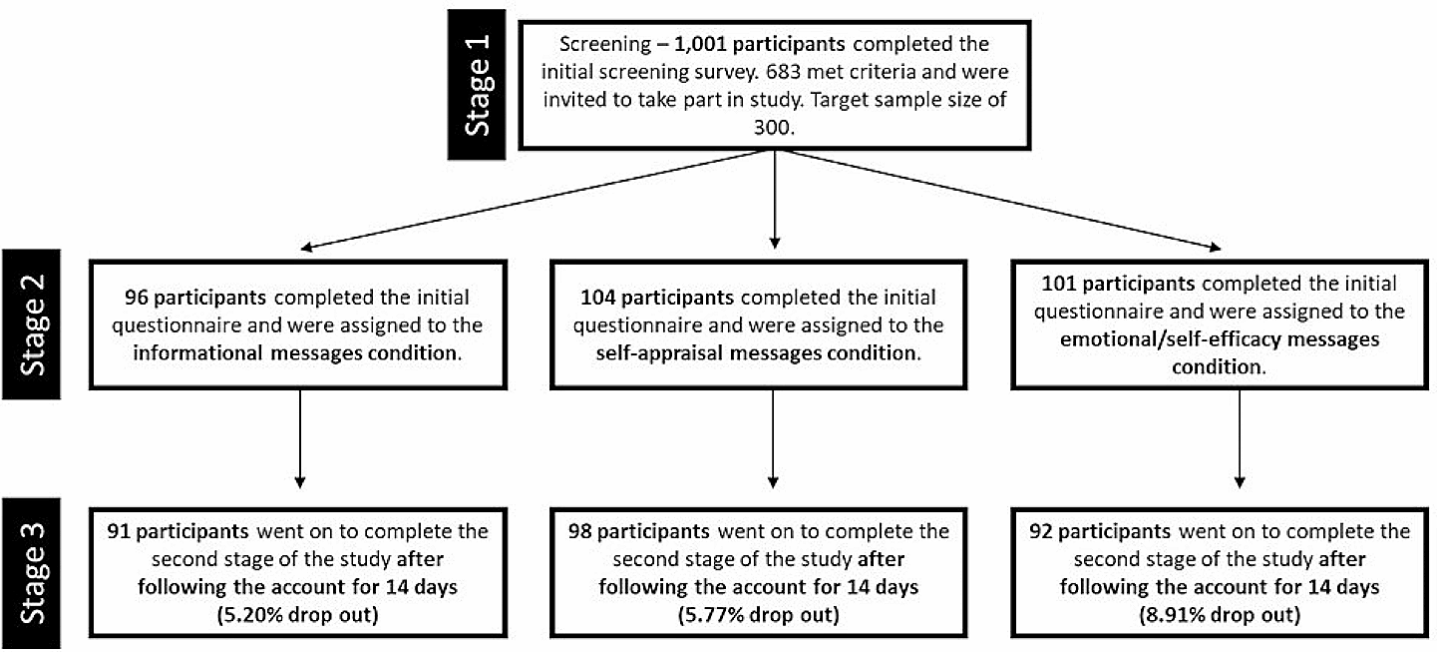
A figure to demonstrate the different stages in participant recruitment, including the number of participants included at each stage and dropout rates

Participants were recruited through the online participant recruitment website Prolific. An initial pre-screening survey was completed on July 9th 2020 by 1,001 individuals to identify participants who had bet on sport at least once a month, on average, and would be willing to follow a social media account set up by the researcher. 683 people met these criteria and were invited to take part in the study, with a target sample size of 300. 301 participants were identified and recruited following the screening process (Stage 1) of the study on July 10th and 11th. Eligible participants were then randomly assigned to a message-condition (Stage 2) and were asked to follow one of three associated social media accounts, set up for the purpose of the study for two-week intervention before completing the study (Stage 3). A total of 281 participants then completed the full messaging-intervention period on July 24th and 25th, with a dropout rate of 6.65% (see Fig. 1 for the full breakdown of participant recruitment by condition of the study). Participants were paid at an hourly rate of £6.60 an hour across the three stages of the study, resulting in participants receiving £0.11 for pre-screening, £1.10 for part 1 and £0.90 for part two. The age of participants within the final sample of 281 ranged from 18 to 64 (mean = 36.60, SD = 10.94), with participants well matched across conditions (see Table 1 for a full breakdown of the categorical and ordinal demographic variables).

**Table 1:** Participant demographic information (Frequency & Percentage) by gender, employment status, highest level of education, ethnicity and relationship status per messaging condition, *N* = 281

	Informational		Self-Appraisal		Emotional/Self-Efficacy		Overall	
	N	%	N	%	N	%	N	%
*Gender*								
Male	74	81.3	80	81.6	70	76.1	224	79.7
Female	17	18.7	18	18.4	22	23.9	57	20.3
*Employment Status*								
Full-time	67	73.6	71	72.4	76	82.6	214	76.2
Part-time	2	2.2	12	12.2	6	6.5	20	7.1
Student	4	4.4	5	5.1	2	2.2	11	3.9
Self-employed	8	8.8	4	4.1	4	4.3	16	5.7
Unemployed	7	7.7	3	3.1	1	1.1	11	3.9
Other	3	3.3	3	3.1	3	3.3	9	3.2
*Highest Level of Education*								
GCSE or Equivalent	8	8.8	6	6.1	6	6.5	20	7.1
A-Level or Equivalent	23	25.3	25	25.5	23	25.0	71	25.3
Undergraduate Degree	39	42.9	46	46.9	39	42.4	124	44.1
Postgraduate Degree	21	23.1	18	18.4	22	23.9	61	21.7
Doctorate	-	-	1	1.0	1	1.1	2	0.7
Other	-	-	2	2.0	1	1.1	3	1.1
*Ethnicity*								
White British	83	91.2	78	79.6	79	85.9	240	85.4
Other	8	8.8	20	20.4	13	14.1	41	14.6
*Relationship Status*								
Single	19	20.9	26	26.5	20	21.7	65	23.1
In a relationship	27	29.7	30	30.6	29	31.5	86	30.6
Married	40	44.0	40	40.8	41	44.6	121	43.1
Divorced	4	4.4	2	2.0	1	1.1	7	2.5
Other	1	1.1	-	-	1	1.1	2	0.7

### Materials

#### Gambling Readiness to Change Questionnaire

The Gambling Readiness to Change Questionnaire (GRCQ) is a nine item questionnaire (Neighbors et al., 2002) that assesses how ready an individual is to make changes to their gambling behaviour. Participants rate statements such as ‘sometimes I think I should cut down on my gambling’ and ‘I have just recently changed my gambling habits’ on a five-point Likert scale from 1 (strongly disagree) to 5 (strongly disagree). A weighted total score was calculated, with a higher score indicating a higher willingness to change gambling behaviour. The GRCQ shows satisfactory reliability (alpha = 0.81) and demonstrates evidence of good convergent validity with gambling outcome measures (Neighbors et al., 2002).

#### Sports Betting Behaviour Questionnaire

A short in-house sports betting behaviour questionnaire was developed which asked participants to report their betting behaviour over the last 14 days. This asked how many days on which they had bet, how many bets they had placed and the total amount of money they had staked. These questions were accompanied by a message which read “on a separate browser or device, please log into all of your gambling accounts in order to answer the next three questions”. This is very important to ensure the accuracy of the data you provide to this study”. This attempted to remove the inaccuracy of self-reporting upon gambling behaviour (Auer & Griffiths, 2017). Finally, a short in-house questionnaire was developed to assess the reach and self-perceived impact of seeing the safer gambling messages on social media. This asked participants how many days they recalled seeing the messages, how many messages they recalled seeing per day and whether they made changes to their betting upon seeing the messages. A text entry box was then used to obtain qualitative feedback on why they did or did not make changes to their behaviour.

#### Safer Gambling Messages

Three Twitter accounts were set up for participants to follow for 14 days. The first account posted informational gambling messages taken from current gambling advice messages used within the gambling industry and several gambling charities (e.g. ‘Remember to only bet with money you can afford to lose #SaferGambling’). The second account posted self-appraisal messages that encouraged participants to reflect upon their own gambling behaviour (e.g. ‘Do you know how much money you have spent gambling in the past 24 hours? #SaferGambling’). The final account posted short emotional responsible gambling videos from the GambleAware ‘BetRegret’ campaign (GambleAware, 2020) alongside messages aiming to increase self-efficacy by giving specific suggestions as to how bettors could make their behaviour safer (e.g. ‘One way in which you can make your gambling safer is to go now and place deposit limits on all of your gambling accounts #SaferGambling’). Tweets were scheduled to be posted in a randomised order at random intervals between 10am and 10pm everyday during the data collection period. Each account posted between four and eight messages each day, with the same number of messages posted to each individual account on any given day. This reflected the type of natural variation which would be present if such a safer gambling strategy was employed in the future.

### Procedure

The study received full ethical approval from [REDACTED FOR ANONYMITY] postgraduate ethics committee (REF − 24,600). Participants accessed the pre-screening survey, which was hosted on Qualtrics, through Prolific. This provided information on how frequently they bet on sport and willingness to follow a social media account for the duration of the study. Those meeting the criteria for the main study were invited to take part in the main study. Participants then completed the initial part of the main study on Qualtrics. After reading the information sheet and providing informed consent, they first completed the demographics questionnaire. They then completed the sports betting behaviour questionnaire, the social media questionnaire, the PGSI and the GRCQ, in that order. Qualtrics then randomly assigned participants to follow one of the three private social media accounts set up for the purposes of the study. Upon following an account, they were informed that they would see messages from the account on their Twitter feed for the next 14 days. Participants were not specifically told to read or interact with the messages, to make the data they provide more naturalistic. After following the accounts for 14 days, participants were invited to complete the second part of the main study through Prolific. On Qualtrics, they were first asked to complete the sports betting behaviour questionnaire, the GRCQ and the short questionnaire assessing the reach and impact of messages seen during the 14 days. Participants were then shown a debrief sheet. In total, the pre-screening survey and the two surveys for the main study took around 20 min to complete. All data and analyses, as well as study materials, are available to view on OSF (https://osf.io/6b389/?view_only=6249d2c6cf2e46e89226282fd3814374 – ANONYMOUS REVIEW LINK).

## Results

### Treatment of Data

After removing participants who did not complete both parts of the study, five missing data points for pre-intervention number of days bet were replaced by inputting the median value for the variable (Kaiser, 2014). Descriptive statistics were then calculated on the reach of the safer gambling messages and two separate one-way independent group ANOVAs were run to assess whether the reach of the messages differed between conditions. Then, to assess the success of the messages on intention to change behaviour and on measures of gambling behaviour (number of bets placed, total money staked, number of days bet), four two-way mixed ANOVAs were carried out. For all six of the ANOVAs carried out, assumption testing was first carried out before proceeding with the analysis and any issues identified are discussed when reporting the analysis.

### Reach of Safer Gambling Messages on Social Media

Descriptive statistics were calculated to investigate the reach of the safer gambling messages delivered on social media (see Table 2). Six participants were removed from the analysis for providing unusable or missing data. On average, participants saw messages from the accounts around every second day, albeit there was large variability within the number of days individuals saw the messages. Additionally, participants saw around two messages from the accounts per day on average. A one-way independent groups ANOVA showed that there was no significant difference in the number of days where messages were seen between conditions, *F*(2, 272) = 1.424, *p* = .243, partial eta squared = 0.10. During assumption testing for the number of messages seen per day ANOVA, five outliers were highlighted as having studentized residual values of larger than three. The analysis was therefore run with and without the outliers, given that the outliers were considered possible responses, and it was found they did not impact upon the overall findings. Therefore, the outliers remained within the analysis. This did mean that the data remained slightly skewed (as seen by normal Q-Q plot), however it is important to note that ANOVA analyses are robust to failures of assumptions of normality with large sample sizes (Driscoll, 1996). It was found that there was no significant difference in the number of messages seen per days between conditions, F(2, 272) = 0.487, *p =* .615, partial eta squared = 0.004.

**Table 2:** Descriptive statistics (M and SD) for number of days where messages where seen and number of messages seen per days, as well as frequencies (N and %) of participants reporting they changed their behaviour in the informational (INF), self-appraisal (S-A) and emotional/self-efficacy (EMO/S-E) messages conditions, as well as overall (OVR)

	INF (*n* = 88)		S-A (*n* = 95)		EMO/S-E (*n* = 92)		OVR (*n* = 275)	
	M	SD	M	SD	M	SD	M	SD
Number of days where messages were seen	7.26	4.51	7.06	4.33	6.21	4.62	6.84	4.49
Number of messages seen per day	2.80	1.60	2.21	1.86	1.97	1.58	2.09	1.68
Changed behaviour in response to messages	N	%	N	%	N	%	N	%
Yes	18	20.5	10	10.5	16	17.4	44	16.0
No	70	79.5	85	89.5	76	82.6	231	84.0

Participants also self-reported whether they had actively made changes to their gambling behaviour after seeing the messages on social media. Overall, most participants (84%) reported that they did not actively make changes to their behaviour after seeing the messages. A chi-squared test of independence was then run to assess whether there was a significant association between study condition and reporting making changes to betting behaviour. Findings revealed no significant association, χ2 (2) = 3.550, *p* = .170.

### Impact upon Behaviour and Intention to Change

A series of four two-way mixed ANOVAs were carried out to assess the impact of intervention stage (pre and post intervention) and message condition (informational, self-appraisal and emotional/self-efficacy) upon three measures of gambling behaviour (number of bets placed, total money staked and number of days bet) and readiness to change gambling behaviour scores. Firstly, within each ANOVA, studentized residuals were examined to check for outliers and were plotted via normal Q-Q plots to assess whether data was normally distributed. For readiness to change, there were only one outlier with a residual of above three and data was normally distributed. However, for the measures of gambling behaviour, there were many outliers with residuals above a value of three and the data was skewed. Each variable of gambling behaviour was therefore transformed using a log10(variable + 1) transformation. After transformation, each variable was approximately normally distributed across the cells of the study design. However, there were still three outliers for bets placed and five for total money staked. Therefore, the ANOVAs for these two variables and readiness to change gambling behaviour were ran with and without the outliers. It was found that there were the main findings were not altered via the inclusion of the outliers and therefore they were left in the analysis due to being considered possible responses. Finally, no tests of sphericity were conducted due to the repeated measures factor only having two levels. Descriptive statistics are presented in Table 3, including both the original variables and the transformed variables.

**Table 3:** Mean (SD) responses on each DV (readiness to change behaviour, number of bets placed, money staked and days bet) by both experimental stage (pre-intervention and during-intervention) and experimental condition (informational, self-appraisal and emotional/self-efficacy), *N* = 281

	Pre-intervention				During-intervention			
	INF (*N* = 91)	S-A (*N* = 98)	EMO/S-E (*N* = 92)	OVR (*N* = 281)	INF (*N* = 91)	S-A (*N* = 98)	EMO/S-E (*N* = 92)	OVR (*N* = 281)
RTC	0.49 (1.19)	0.59 (1.09)	0.50 (1.07)	0.53 (1.12)	0.78 (1.37)	0.76 (1.31)	0.65 (1.27)	0.73 (1.31)
Bets Placed	17.07 (40.23)	27.60 (81.77)	15.17 (20.52)	20.12 (54.81)	14.18 (29.33)	19.84 (40.73)	13.22 (20.39)	15.84 (31.54)
Money Staked (£)	201.61 (841.95)	203.60 (583.34)	174.19 (581.76)	193.33 (675.22)	170 (719.02)	340.76 (2061.43)	179.37 (837.97)	232.79 (1368.49)
Days Bet	5.32 (3.98)	6.47 (4.45)	6.28 (4.36)	6.04 (4.29)	5.09 (4.19)	5.61 (4.82)	5.71 (4.33)	5.47 (4.46)
Bets Placed (Transformed)	0.95 (0.46)	1.02 (0.52)	1.00 (0.41)	0.99 (0.47)	0.85 (0.50)	0.90 (0.58)	0.90 (0.45)	0.88 (0.51)
Money Staked (Transformed)	1.64 (0.60)	1.65 (0.69)	1.68 (0.63)	1.66 (0.64)	1.50 (0.74)	1.48 (0.85)	1.55 (0.69)	1.51 (0.76)
Days Bet (Transformed)	0.72 (0.28)	0.79 (0.29)	0.78 (0.28)	0.76 (0.28)	0.67 (0.33)	0.68 (0.38)	0.73 (0.31)	0.69 (0.34)

There was homogeneity of variance within each cell of the design for each DV (*p* > .05), except for ‘during intervention’ for bets placed, as assessed by Levene’s test of homogeneity of variance. There was also homogeneity of covariances for each DV, as assessed by Box’s test of equality of covariance matrices (*p* > .001). There was no statistically significant interaction between condition and intervention stage upon bets placed, F(2, 278) = 0.274, *p* = .761, partial eta squared = 0.002, 90% CI [0, 0.123], money staked, F(2, 278) = 0.215, *p* = .806, partial eta squared = 0.002, 90% CI [0, 0.011], number of days bet, F(2, 278) = 1.933, *p* = .147, partial eta squared = 0.014, 90% CI [0, 0.040], and readiness to change gambling behaviour, F(2, 278) = 0.523, *p* = .593, partial eta squared = 0.004, 90% [0, 0.006].

There was also no significant main effect of condition on bets placed, F(2, 278) = 0.476, *p* = .622, partial eta squared = 0.003, 90% CI [0, 0.018], money staked, F(2, 278) = 0.150, *p* = .860, partial eta squared = 0.001, 90% CI [0, 0.008] number of days bet, F(2, 278) = 1.003, *p* = .368, partial eta squared = 0.007, 90% CI [0, 0.027] or readiness to change gambling behaviour, F(2, 278) = 0.177, *p* = .838, partial eta squared = 0.001, 90% CI [0, 0.009].

However, there was a significant main effect of intervention stage on bets placed, F(1, 278) = 31.743, *p* < .001, partial eta squared = 0.102, 90% CI [0.053, 0.161], money staked, F(1, 278) = 27.232, *p* < .001, partial eta squared = 0.089, 90% CI [0.043, 0.145], number of days bet, F(1, 278) = 20.359, *p* < .001, partial eta squared = 0.068, 90% CI [0.028, 0.120], and readiness to change gambling behaviour, F(1, 278) = 11.787, *p* = .001, partial eta squared = 0.041, 90% CI [0.011, 0.085]. Each measure of gambling behaviour showed a reduction during the intervention compared to pre-intervention, whilst readiness to change gambling behaviour scores increased from pre to during intervention (see Table 3).

### Qualitative Feedback on Impact of Messages

To gain a deeper understanding of why participants did or did not make changes to their betting behaviour during the 14 days where they were asked to follow the social media accounts, participants were asked to provide a written explanation as to why they did or did not make changes. In total, 260 participants (93% of final sample) provided feedback and each response was coded individually for the main reason as to why they either did or did not change their behaviour. Once each response had been coded, codes were then grouped together and highlighted five main reasons as to why participants did not make changes to their behaviour after seeing the messages, as well as two main reasons identified as to why messages did lead to a change in behaviour. There was also a small number of responses (*n* = 15) which were excluded from any of the final seven categories for responses due to either their response being unclear or their response not being relevant to the question asked. The number of responses within each category was then calculated. Given the lack of significant main effect of condition or interaction effect, it was decided that there would be no comparison of the frequency of reasons given between conditions. Please see Table 4 for a description of each of the categories, their frequency and examples.

**Table 4:** Categories for reasons why messages led to participants changing their behaviour or not, their frequency and example quotes, *N* = 260

Category	Description	Example
No need to change gambling behaviour (*n* = 108)	Participants stressed that their gambling was seen a recreational activity carried out for fun and that they only bet with low stakes and therefore they had no reason to change their behaviour after reading the messages. Additionally, some participants stated the reason that the messages had no impact was because they were not problem gamblers, suggesting they were seen as a reaction measure for those experiencing harm	*“The messages seemed to be aimed at getting people to gamble less and more responsibly. I would already consider myself to be a sensible, modest gambler”*
		*“My gambling isn’t a problem, those messages are for problem gamblers, not me”*
Content of messages (*n* = 29)	Participants noted that the specific content of the messages was not relevant to them or their gambling behaviour. Participants stated that they had seen similar messages before taking part in the study and that this limited the potential impact the messages had. The messages were also said to not be persuasive enough and this limited the impact they could have upon betting behaviour	*“The messages, though helpful, weren’t really relevant to my current behaviour. Many tweets referred to either using deposit limits (which are already in place) or avoiding gambling whilst under the influence of alcohol (I rarely drink). Good messages, but less relevant for me in particular”*
		*“The information was very general and nothing I don’t see regularly, I have the same messages emailed to me by bookmakers etc and have read them all already dozens of times”*
Messages were not seen or were seen at the wrong time (*n* = 24)	Several participants stated they didn’t see messages from the accounts during the 14 days they were asked to follow the accounts. Reasons given for this included got lost within their participant’s twitter feeds, not using social media very often, ignoring the messages or that the messages failed to grab their attention. Some participants saw messages during times where they would were not gambling and therefore they did not have an impact on their behaviour	*“Did not see any messages from the account, my twitter feed was saturated with other content”*
		*“They didn’t catch my attention enough in the feed”*
Personal factors (*n* = 21)	A range of personal factors were also put forward to explain why messages had limited impact. Such reasons included: not wanting to place a bet during the 14-day period, not wanting to make changes to their behaviour, already following the advice given in the messages and gambling behaviour being at a clinical level and therefore being hard to change/	*“I dont gamble much and there was no occasions I felt like betting on anyway”*
		*“I felt that I had already put changes in place and cut my gambling down to a lower level”*
Participant’s betting strategies rendered messages irrelevant (*n* = 18)	Participants argued that their specific betting strategies reduced the risk associated with betting and therefore they did not need to follow the advice given in the messages. Examples of this included; only taking part in matched betting, having systems in place to monitor their gambling, only gambling in response to marketing and that their gambling is profitable	*“my gambling is 99% Matched Betting, so although I place a large number of bets and bet significant sums, it is done with guaranteed profit in mind. I VERY rarely bet in the traditional sense and although i use all the tools of a gambler, I am not gambling as I am not losing (Ive made around £2000 in the last 18months)”*
		*“No need to make any changes, I track every bet in a spreadsheet and do a lot of research”*
Encouraged reflection or action upon gambling behaviour (*n* = 37)	Messages encouraged participants to reflect upon certain aspects of their gambling behaviour which the messages commented upon. Examples of this included: helping them realise they were placing bets due to boredom, following limit setting advice, encouraging them to act on concerns about behaviour and reminding them of the issues gambling has caused them	*“I pondered on some messages I saw on the account and decided to follow one of the messages that says I can set deposit limits on my gambling account. I loved that message, i did it and it has helped me immensely”*
		*“It was already something I was thinking about, but seeing the Twitter only solidified my concerns. I took timeouts on my gambling accounts”*
Raised awareness of the problems gambling can cause (*n* = 7)	The other reason given by a small number of participants for making changes to behaviour after seeing the messages was due to the message increasing awareness of the problems gambling can cause. More specifically, participants stated that the messages made them think about what could happen if they let their own gambling behaviour spiral out of control	*“Because it makes me worried about getting addicted back to gambling and I won’t have no control over it, so seeing these messages would help me control my addiction better and help improve my life”*

## Discussion

### Summary of Findings

The current study assessed the impact of receiving safer gambling messages on social media upon betting behaviour and readiness to change gambling behaviour, as well as assessing effectiveness of different message types. It was predicted that participants in the self-appraisal and emotional self-efficacy message conditions, but not those in the informational condition, would show reduced betting activity during the 14-day study period where they were receiving the messages, and an increased readiness to change gambling behaviour after the 14 days. Participants reported reduced betting behaviour during the study period and increased readiness to change at the end of the study period, regardless of which account they were asked to follow. Therefore, the findings only provide partial support for the hypotheses as the changes in behaviour identified within the self-appraisal and emotional self-efficacy conditions were also identified within the control condition of informational messages. As such, whilst findings may suggest an impact of receiving safer gambling messages on social media regardless of message type, changes in behaviour identified may be a result of knowing behaviour was being tracked rather than the impact of the messages.

Additional information collected within the study offers further insight into the main findings. In terms of reach, participants saw messages from the accounts on every second day and saw two messages a day. This was consistent across conditions of the study. However, only 16% of participants reported actively choosing to make changes to their gambling after seeing a safer gambling message on social media. Qualitative data provided an insight into why participants chose not to make changes to behaviour, with the most identified reasons being that they saw no need to change their behaviour or that the content of the messages was not relevant to them. Of those who did change their behaviour, they stated that messages encouraged them to reflect upon their behaviour and increased their awareness of issues that gambling can cause.

### Contribution to Existing Literature

The finding that participants displayed reduced betting behaviour during the 14-day period where they were asked to follow the account provides some initial evidence that receiving safer gambling messages on social media may impact upon behaviour, with a medium effect size observed for each behavioural measure. This is supported by increased readiness to change gambling behaviour scores at the end of the 14-day period, albeit this was only a small effect. This appears to support research from other areas of public health that suggests social media can be an effective medium to produce behavioural change (Laranjo et al., 2015). The medium effect size observed is similar to the average effect size calculated in a recent meta-analysis on the impact of pop-up messages during gambling sessions (Bjørseth et al., 2021). This suggests messages received outside of a betting session have the potential to have a similar impact in reducing betting behaviour as in-play messages for machine gambling, highlighting that both types of messages are needed to help reduce gambling-related harm.

However, doubt is raised over the changes in behaviour observed being the result of message content due to the observed reduction in behaviour in the informational messages condition, given informational safer gambling messages have consistently been demonstrated to have a lack of impact upon gambling behaviour (Cloutier et al., 2010; Gainsbury et al., 2015). One potential explanation for this is that informational messages were not appropriate as a control condition and do in fact have the potential to instigate changes in behaviour. This may be because messages within the current study were implemented outside of an active session, whereas previous studies have largely looked at their impact when delivered whilst participants were actively gambling. This could have allowed messages to become embedded within the daily routines of participants whereby the prolonged exposure to messages, alongside the absence of high emotional states present during gambling sessions (Sohn et al., 2015), allows bettors to process the messages in a way which initiates behavioural change. An alternative explanation is that participants reduced their behaviour during the period where they followed the accounts due to demand characteristics (Orne, 1962) or as a direct impact of knowing their behaviour would be monitored over the two weeks, regardless of the account they were asked to follow. Therefore, future research should aim to clarify this by replicating the current study with a control condition where participants do not receive any safer gambling messages.

The current study supported previous research in demonstrating the potential for social media health messages to have a high reach within the intended population (Gough et al., 2017), with messages seen on average once every other day. However, the variability in the number of days messages were seen was large. This suggests other factors may impact upon message visibility for the intended audience. Whilst such factors were not assessed within the current study, it makes logical sense that factors such as the number of accounts an individual follows and the time they spend on social media would impact upon the likelihood of seeing messages. There is also the potential that not all posts made from the accounts made it into the Twitter feed of every participant. One potential solution for increasing the reach of messages would be to invest finances into promoted messages on social media in order to reach a larger number of individuals and to give tweets increased prominence upon an individual’s timeline. Alternatively, the frequency of messages posted could be increased.

Several reasons were put forward by participants as to why they did not change their behaviour during the study. The most common reason was that they did not see any need to change their behaviour as they did not encounter problems from their betting. This finding can be explained by the previously covered Protection Motivation Theory [PMT] (Rogers, 1975), which explains that individuals make a judgment on their perceived vulnerability and susceptibility when viewing a health message. Therefore, if participants within the study did not view themselves as being vulnerable to developing gambling problems, this would explain why they did not actively look to change their behaviour after seeing the messages. Additionally, the fact that many participants commented upon the fact they did not have problems with gambling highlights that they see such safer messages as a reactionary measure rather than a general principle to follow. This highlights an inherent issue with currently used safer gambling approaches, which focus upon personal responsibility (Blaszczynski et al., 2011). Such approaches rely on individuals being able to identify when their behaviour is causing them harm and then to take the appropriate measures to reduce such harm. If individuals do not recognise the harm which their behaviour may be causing them, or feel as though negative consequences they experience are not serious, then they are unlikely to perceive the messages or their content as relevant.

However, those individuals who did make changes to their betting behaviour after seeing the messages mainly stated that this was due to messages making them reflect upon their gambling behaviour. Interestingly, this was the case across conditions and not just in the self-appraisal condition, where participants were encouraged to reflect upon certain aspects of their behaviour. This, along with the non-significant interaction effect, contrasts with findings on the impact of messages during in-play gambling sessions which have shown self-appraisal messages to be more useful than informational messages in encouraging changes in behaviour (Gainsbury et al., 2015; Harris & Parke, 2016). This suggests that outside of gambling sessions, the impact of safer gambling messages may rely upon other factors than the type of message an individual sees. The use of personalised feedback messages has received some support within the literature when delivered within gambling sessions (Auer & Griffiths, 2013; Kim et al., 2014; McGivern et al., 2019), however this would be considerably more difficult to work into social media messaging due to needing information on an individual’s behaviour in order to provide personalised feedback. As such, future research should focus upon exploring the personal factors which impact upon the successfulness of different types of safer gambling messages.

### Evaluation of Current Study

The current study has several strengths. Firstly, the study obtained a more objective measure of betting behaviour than in a range of previous studies by asking participants to log into their online gambling accounts to view their accounts before reporting their betting behaviour. Whilst this does not completely remove the issue of participants providing false information, it does counter the memory biases which have been previously identified with asking participants to remember how much they have gambled (Auer & Griffiths, 2017; Braverman et al., 2014). It also gives the benefit of requiring participants to report their behaviour at less frequent intervals than in other studies which have sought to counter the impact of memory biases (Browne et al., 2019). Additionally, the inclusion of a single qualitative question at the end of the study was useful in obtaining a deeper understanding as to participant’s thoughts upon the messages and why they did or did not encourage them to change behaviour. This highlighted some wider issues around personal responsibility approaches to safer gambling, whereby messages are often seen as not being relevant by gamblers. As such, this highlights the fact that the use of safer gambling messages to prevent escalating gambling problems may be limited by the misinterpretation of the purpose of such messaging.

However, one limitation of the current study is that data on participant’s betting was only collected for the two weeks prior to following the account and the two weeks whilst they were following the account. If participants had been asked to follow the account for a longer period, or if data had been collected post following the accounts, this may have allowed for further insight into the significant main effect observed in the study. This is because participants may be less likely to show demand characteristics over a longer time period. As such, there would be more confidence in the main effect observed being a result of receiving the messages as opposed to reducing behaviour as a result of knowing they were taking part in a study. However, increasing the period between the different parts of the study would likely have resulted in a larger dropout rate which also has the potential to impact negatively upon findings if there is a systematic pattern to the types of individual who are more likely to drop out (Wolke et al., 2009). Another potential limitation of the current study is that it could be argued that watching the emotional messages requires more investment from participants than either of the other two message types. This is because emotional messages were in the form of a video whereas the other messages were just in written text. Whilst emotional messages were seen as commonly as the other messages, there was no data collected which indicated whether participants were watching the videos provided or not. As such, it may be that the emotional condition did not lead to greater reductions in behaviour simply due to participants not engaging with the emotional video content.

### Future Directions

To build upon the findings of the current study, future research should focus on replicating the current study with an additional control condition. This would address whether the main effect observed within the current study was due to messages having an impact directly or due to a placebo-like effect whereby participants moderated behaviour simply as a result of knowing they would have to report their behaviour. For example, if a fourth ‘waiting list’ condition was included whereby participants do not receive any messages over the two-week period, this addition to the study would be able to assess whether a similar reduction in behaviour was present and therefore whether changes in behaviour were due to receiving messages or not. Alternatively, future research could take a longitudinal approach in order to assess the impact of following the accounts over a longer period. This would limit the possibility of participants modifying their behaviour as a result of knowing their behaviour was being observed as this would be much harder to maintain over a longer time period.

## Conclusions

The current study assessed the reach and effectiveness of delivering safer gambling messages to frequent gamblers on social media. It also assessed whether one type of safer gambling messaging was particularly effective in helping individuals make positive changes towards their betting behaviour. The current findings suggested that social media safer gambling messages may have led to reductions in betting behaviour during the period where participants were asked to follow the account. However, the lack of a significant interaction effect creates doubt over this given that the control group of those receiving informational safer gambling messages, which have been shown in previous research to have no impact upon gambling behaviour (Cloutier et al., 2010; Gainsbury et al., 2015), demonstrated a similar reduction in behaviour to the two experimental conditions. These doubts are further exacerbated by the fact that less than one in five participants within the study reported actively choosing to make changes to their behaviour after seeing the messages, albeit the impact of the messages could be more subtle than this. Qualitative feedback suggested the most common reason for not changing behaviour was due to not seeing any need to change their behaviour, highlighting a limitation of personal responsibility approaches to safer gambling. Future research should aim to offer further insight into the current findings by replicating the study with an additional control condition or by asking participants to follow the Twitter accounts for a longer time-period.

## Electronic Supplementary Material

Below is the link to the electronic supplementary material.

Supplementary Material 1
