# Supplementary material for: Exploring the Impact of Safer Sports Betting Promotion on Social Media: An Experimental Study
Source: J Gambl Stud. 2024 Jan 19;40(3):1085–102. doi: 10.1007/s10899-024-10280-z (PMC11390894; doi:10.1007/s10899-024-10280-z)
Supplement: Supplementary file 1 — Supplementary Material 1 [file 10899_2024_10280_MOESM1_ESM.docx]

**Scott Houghton**

Northumbria University

Department of Psychology

Tel: (+44) 07983092958

[scott2.houghton@northumbria.ac.uk](mailto:scott2.houghton@northumbria.ac.uk)

21^st^ *April 2023*

Dear Professor Grant,

Please find enclosed our research article with the working title: *Exploring the impact of safer gambling promotion on social media: An experimental study*, which we would like to submit for consideration to Journal of Gambling Studies. For the purposes of Open Access, the author has applied a Creative Commons Attribution (CC-BY) License to any Author Accepted Manuscript version arising.

Harms arising from gambling are a major public-health issue within the United Kingdom. Previous research looking to reduce gambling-related harms has mostly looked at implementing safer gambling messages during gambling sessions, with little research exploring the effectiveness of such messaging delivered outside of sessions. Social media health campaigns have shown success in other areas of public health, however have not yet been employed within gambling research.

The current study aimed to explore whether exposing regular sports bettors to safer gambling messaging on social media had an impact upon gambling behaviour and if so, what type of messaging was most effective. We feel that the findings of this study highlight the high reach of safer gambling messages on social media and the potential impact they could have upon behaviour. This adds to a growing body of literature on harm reduction strategies for gambling and emphasises the importance of future research within this area.

Yours sincerely,

Dr Scott Houghton, BSc., MRes, PhD.

(Signed on behalf of both authors.)
